# Knowledge and confidence of South African health care providers regarding post-rape care: a cross-sectional study

**DOI:** 10.1186/1472-6963-13-257

**Published:** 2013-07-03

**Authors:** Ruxana Jina, Rachel Jewkes, Nicola Christofides, Lizle Loots

**Affiliations:** 1 School of Public Health, Faculty of Health Sciences, University of the Witwatersrand, Johannesburg, Gauteng, South Africa; 2 Gender and Health Research Unit, South African Medical Research Council, Johannesburg, Gauteng, South Africa

**Keywords:** South Africa, Rape, Training, Knowledge, Health providers

## Abstract

**Background:**

In South Africa, providers are trained on post-rape care by a multitude of organisations, resulting in varied knowledge and skills. In 2007, a national training curriculum was developed and piloted in the country. The objectives of this paper are to identify the factors associated with higher knowledge and confidence in providers at the commencement of the training and to reflect on the implications of this for training and other efforts being made to improve services.

**Methods:**

A cross-sectional study using questionnaires was conducted. Providers who attended the training provided information on socio-demographic background, service provision, training, attitudes, and confidence. Knowledge was measured through multiple choice questions. Bi-variable analysis was carried out in order to test for factors associated with high knowledge and confidence. Variables with a p value of <0.20 were then included in backward selection to develop the final multivariable models.

**Results:**

Of the 124 providers, 70% were female and 68% were nurses. The mean age of the providers was 41.7 (24 – 64) years. About 60% of providers were trained in providing post-rape care. The median percentage knowledge score was 37.3% (0% - 65.3%) and the median percentage confidence score was 75.4% (10% - 100%). Having a more appropriate attitude towards rape was associated with higher knowledge, while older providers and nurses had lower odds of having high knowledge levels. Working in a crisis centre in the facility, having examined a survivor in the last 3 months, and seeing more than 60% of survivors who came to the facility were associated with higher confidence. Higher confidence was not associated with greater knowledge.

**Conclusion:**

The study indicated that although confidence was high, there was poor knowledge in providers, even in those who were previously trained. Knowledge seems to be critically dependant on attitude, which highlights the need for educating providers on rape and the seriousness of the problem. There is a need to train more providers in post-rape care in country, and to ensure that training is comprehensive, and that providers who are trained remain knowledgeable and skilled in current best practices.

## Background

In South Africa, the majority of rape survivors are cared for in the public sector at primary health care level. Post-rape care should be provided by trained doctors and nurses [1], yet studies have shown that not all providers receive training. Most are only trained at an undergraduate level, and such training often focuses only on the forensic aspects of post-rape care, thus neglecting the medical needs of patients [2,3]. A number of training programmes have been developed, ranging from undergraduate and postgraduate programmes to informal in-service training initiatives [2,4] and in 2007 the National Department of Health commissioned the development of a standardized training curriculum for health care providers as piecemeal training had resulted in varied levels of knowledge and skills among providers [2,5].

The National Department of Health aimed to use the curriculum to equip doctors and nurses to comprehensively provide post-rape care. The curriculum was developed by a number of local experts under the coordination of the Gender and Health Research Unit of the Medical Research Council. This work was based on reviews of both national and international training programmes. The newly developed curriculum finally comprised of ten days of interactive training and a one day practicum, covering all aspects of post-rape care including epidemiology, human and sexual rights, communication, medical management of the survivor, examination and evidence collection, follow-up care, laws and court procedures, monitoring and evaluation of services, and vicarious trauma. Vicarious trauma is known as compassion fatigue that is felt by providers who work with traumatized patients and can potentially affect the physical and mental well-being of the provider [6]. The curriculum was tested in a pilot programme that was held in eight of the nine provinces in the country.

In developing the curriculum, a number of issues came to the forefront. Firstly, what is the current knowledge and confidence of health providers working in post-rape care and would the curriculum improve this, what are the factors that influence knowledge, confidence and care offered by providers, and finally, which providers should we be training to work in post-rape care services?

A review of international post-rape training programmes showed that there were marked differences in the courses offered to providers [7-16], with lengths ranging from a 45 minute lecture to medical students [15] and 2 hours training on clinical forensic medicine in the United States of America (USA) [8], to a 6 month rotation in forensic medicine for emergency room physicians in Australia [13]. The nursing programmes especially the Sexual Assault Nurse Examiner (SANE) programmes in the USA [9,10] and Canada [9,11] were found to be the most established with a 5 – 7 day didactic training programme followed by clinical exposure in a practical setting. The content of the courses have also varied, with a focus on the examination of survivors and evidence collection. Some of these programmes have reported on improvements in provider knowledge and skills after training but these studies were conducted with small groups in limited settings and did not include a mixed group of providers in a national programme [7,10,15,16]. No studies were found from developing, low resourced settings. Furthermore, no studies were found that investigated other factors influencing knowledge, skills and confidence in providers managing post-rape care survivors.

Very few studies have looked at the providers working in post-rape services. A review of the SANE programmes has shown some positive findings with SANEs providing more comprehensive care and examinations than what was traditionally provided by the hospital emergency department [16,17], that evidence collection kits were better completed by SANEs than by non-SANE nurses or physicians, that police laid more charges and there were higher conviction rates with longer average sentences in SANE cases compared to pre-SANE cases, and that there was an improvement in the relationship between the medical and legal professionals after SANE programmes were implemented [17]. A previous study with women in South Africa on experiences and preferences for post-rape care reported a preference for doctors over nurses. While the women in this study also preferred to have a female provider [18], there were mixed findings regarding this in a review [19] focusing on women’s expectations and experiences with interpersonal violence services. What is very clear is that survivors wish to have a non-judgemental, compassionate and sensitive provider [18,19].

The objectives of this paper are thus to identify the factors associated with higher knowledge and confidence in health care providers working in post-rape care services at the commencement of training and to reflect on the implications of this for training and other efforts being made to improve services.

## Methods

A cross-sectional study was undertaken with data collected during four training sessions that were held from February to May 2008. One hundred and fifty-two providers from eight provinces attended the training. The Western Cape provincial Department of Health declined to participate in the study. Health care providers who were selected by provincial managers to attend the training were either doctors who had completed internship or professional nurses with midwifery qualifications. All providers had some previous exposure to post-rape care services.

Providers who underwent the pilot training completed self-administered questionnaires. These had questions on social and demographic characteristics, the manner in which rape services were provided, the workload in relation to post-rape care services, prior training on counselling and post-rape care services, confidence in providing post-rape care, gender attitudes, attitudes about issues related to rape, and levels of empathy. The questionnaire made use of questions that had previously been tested in research conducted by the Gender and Health Research Unit, including an instrument used in a situation analysis of sexual assault services (1), and a questionnaire used in a survey of men’s health and relationships (11). For the purposes of this study, only data from the questionnaires completed prior to the interactive training component are presented.

Socio-demographic and background information was collected on sex, age, rank, and the province where the health care provider was working, the type of facility where they were working, and the number of years they had been working in total and at the current facility. Data was also collected on the proportion of rape survivors seen by the provider and whether the provider had seen a rape survivor and completed the necessary legal documentation (J88 form) in the last three months. Information on where undergraduate training occurred and whether the provider received further training on post-rape care and counseling was obtained.

A rape attitude score (Cronbach’s alpha 0.82) based on 21 statements [20] was obtained using a four level Likert response from ‘strongly agree’ to ‘strongly disagree’. Examples of statements used in the score included: “many rapes happen because women lead men on”, “sex workers cannot really be raped”, and “a woman who has been raped has a serious medical problem”. The Likert scores were added for each of the statements to develop a total rape attitude score that could range from 21 to 84, with a higher score indicating a more appropriate attitude to rape. A gender attitude score (Cronbach’s alpha 0.76) was similarly developed using the same Likert responses, but based on another 21 statements [21]. Three examples of statements used for this score are: “a man should have the final word about decisions in his home”, “a woman’s most important role is to take care of her home and cook for her family”, and “there are times when a woman deserves to be beaten”. Again the score could range from 21 to 84, with a higher score indicating that the provider was more gender sensitive. These scores were constructed from work done by Burt [20], and Pulerwitz and Barker [21].

An empathy score (Cronbach’s alpha 0.77) was also calculated using four statements, that were adapted from work done by Abbey *et al.*[22]. These had a five level Likert response scale ranging from ‘doesn’t describe me well’ to ‘describes me well’. Two statements included here were “when I see someone being taken advantage of, I feel protective toward them”, and “I often have tender, concerned feelings for people less fortunate than me”. The Likert scores for each statement were added to develop a score with a range from 4 to 20. A higher score indicated that the provider had greater empathy.

Men were also asked about perpetration, and women about experience, of intimate partner violence using a version of the WHO violence against women instrument (12). This had been adapted for use with men in the Eastern Cape (11, 13). It included two questions related to physical abuse, one to emotional abuse, and four to sexual abuse. For example, female providers were asked about being slapped, pushed, shoved, hit, kicked or having something thrown at them by a partner. They were also asked about whether a partner had ever threatened to hurt them or use a weapon against them. They were then asked about whether they had ever been tricked into having sex with a man, whether they had ever agreed to have sex with a man after he told lies, threatened to end the relationship, or pleaded; whether they had ever had sex when they had not consented or were forced, and whether they had ever slept with a man or boy when they were too drunk to say whether they wanted to or not.

Knowledge was assessed using 75 multiple choice questions covering the various aspects of post-rape care. These covered all aspects of the curriculum material and were developed and reviewed by staff at the Gender and Health Research Unit. The questions were piloted on a group of ten medical doctors, after which some changes were made. In addition, nine questions that had a particularly high failure rate through all pilot sessions were excluded from the final data analysis, resulting in a total of 66 questions.

Health care providers reported on their confidence in providing ten aspects of post-rape services, using a score of 1 to indicate ‘no confidence’ and 10 to indicate ‘total confidence’ in providing the service. Questions covered examination, evidence collection, and appearing in court. Both the knowledge and confidence scores were converted into percentages and categorized. Providers with a percentage score of 50 or more were considered to have high levels of knowledge and those with a percentage score of 80 or more were considered to have high levels of confidence. These cut points were based on the mean scores for knowledge and confidence levels.

Data was entered and analysed using Stata 10. Descriptive statistics were first conducted on the baseline characteristics of the health care providers in terms of background, experience, knowledge and confidence, after which bi-variable analysis was carried out in order to test for factors associated with high knowledge and confidence separately. Variables with a p value of <0.20 were included in the candidate models and backward selection was used to develop the two final multivariable models on factors associated with high knowledge and confidence. In the final models, variables were retained with a p value of less than or equal to 0.05.

Ethics approval was obtained from the Human Research Ethics Committee: (Medical) of the University of the Witwatersrand (Approval No. M071140). The health care providers were given an information sheet and were asked to sign informed consent when they returned the completed questionnaire. Participation in the study was thus optional and utmost care was taken to ensure that the anonymity of the study participants was maintained.

## Results

In total, 124 health care providers (81.6% of those trained) completed pre-training questionnaires. The mean age of the providers was 41.7 years with a range from 24 to 64 years. The majority of providers trained were female (70.2%) and professional nurses (68.3%). Of the 39 doctors who were trained, six were completing their community service year, which is a compulsory second year of working in the public sector after completion of medical school (Additional file 1: Table S1). Forty-seven of the 87 female providers (56.6%) had experienced some form of intimate partner violence in their lifetime, while 13 of the 37 male providers (36.1%) had perpetrated acts of intimate partner violence. Sexual abuse was the most common form of abuse experienced (38.5%) and perpetrated (30.6%), followed by physical abuse (37.4% and 22.2%). About a third of the providers felt that rape was not a serious medical problem (n=34, 28.8%).

The majority (76.0%) of providers were working at the primary health care level (clinics, community health centres and district hospitals) (Additional file 1: Table S2). Just under half of the providers (44.4%) worked in facilities that had a crisis centre. The providers had a median of 14.5 years of experience with a range of 1 to 36 years, and the median number of years at the current facility was 5 years (range 0 – 36 years). About half of the providers (49.6%) had examined a survivor and had completed a J88 form in the three months preceding the training. About 60% of providers had been trained in providing post-rape care, while 79% reported having been trained in counselling. The levels of knowledge were low with a median percentage score on the knowledge assessment of 37.3% and a range of 0% to 65.3%. A quarter of the providers (n=29) scored 50% or more. Providers scored less than 50% on questions related to communication, examination and evidence collection of adult survivors, management of child survivors, mental health care, prevention and management of pregnancy post-rape, and vicarious trauma (Additional file 1: Table S3). Confidence was much higher, as providers had a median confidence percentage of 75.4% with a range of 10% to 100%. A third of the providers (n=33, 33.0%) had confidence levels of 80% or more (Additional file 1: Table S2).

Sixty one percent of providers with high levels of knowledge were doctors compared to 24% of providers with low knowledge (Additional file 1: Table S1). On the other hand, 76% of providers with low knowledge were nurses compared to 39% with high knowledge (p value 0.000). Younger providers (mean of 37 years versus 43 years) were found to have significantly higher levels of knowledge (p value 0.002). Significant interprovincial differences were noted in knowledge levels (p value 0.025) with KwaZulu-Natal and Free State having a high proportion of providers with high levels of knowledge (28% and 21% respectively) compared to the proportion from these provinces among those who had low levels of knowledge (14% and 5% respectively). Disproportionately few providers in the Eastern Cape and North West Provinces were among those who had high levels of knowledge (7% and 0% respectively), compared to the proportion among those who had low levels of knowledge (20% and 14% respectively). The providers with a higher rape attitude score were also found to more often have significantly higher levels of knowledge (p value 0.027). None of the providers who had been working at their current facility for more than 15 years had high levels of knowledge, but this group constituted 19% of those who had low levels of knowledge; while 74% of those who were in the facility for less than 10 years had high levels of knowledge compared to 60% who had low levels of knowledge (p value 0.030) (Additional file 1: Table S2). Finally, providers who had examined a survivor and completed a J88 form in the last three months were found to have higher levels of knowledge (72% vs. 43% of those who had not, p value 0.005).

The pattern of distribution of provider confidence differed from that of knowledge (Additional file 1: Table S1). In the Free State and Mpumalanga 24% and 21% of providers were found to have high levels in confidence, compared to 8% and 8% of providers with low levels of confidence. Limpopo contributed only 6% of providers with high levels of confidence, and the North West, none, compared to those with low levels of confidence (13% and 11% respectively) (p value 0.034). Providers who worked in a facility with a crisis centre more often had high confidence (67% vs. 34% of those not doing so, p value 0.002) as had those who had examined a survivor and completed a J88 form in the last three months (73% vs. 43%, p value 0.006) (Additional file 1: Table S2). Trained providers were found to have high levels of confidence (79% vs. 53% not trained, p value 0.013) although no association was found with higher levels of knowledge (Additional file 1: Table S2). In addition, a high confidence level was not found to be associated with a higher level of knowledge; 33% of providers with high knowledge had high levels of confidence, as did 31% of the providers with low knowledge (p value 0.810) (Additional file 1: Table S2).

The following variables were tested in the final multivariable model looking at factors associated with higher levels of knowledge: sex, age, rank, experience or perpetration of interpersonal violence, rape attitude score, total time in services, examination of a survivor and completion of a J88 form in the last three months, and having been previously trained on counselling (Additional file 1: Table S4). With the final multivariable model, it was found that older providers (OR 0.94, 95% CI 0.88 – 1.00) and nurses (OR 0.40, 95% CI 0.23 – 0.69) had lower odds of having high levels of knowledge while providers with a more appropriate rape attitude score (having a higher rape attitude score) had a greater odds of having high levels of knowledge (OR 1.10, 95% CI 1.01 – 1.19) (Additional file 1: Table S4).

In terms of confidence levels, providers who worked in a facility with a crisis centre, those who had examined a survivor and completed a J88 form in the last three months, those who had been trained on providing post-rape care in the past and the proportion of survivors seen by the provider met the criteria to be tested in the multivariable model. In the final multivariable model, providers who had worked in a facility with a crisis centre (OR 3.08, 95% CI 1.19 – 7.99) and those who had examined a survivor and completed a J88 in the last three months (OR 3.57, 95% CI 1.29 – 9.89) had a greater odds of having higher levels of confidence. Providers who had seen more than 60% of survivors who came to the facility were also found to have greater odds of having high confidence compared to providers who had seen less than 20% of survivors (OR 4.31, 95% CI 1.23 – 15.10). Previous training was not found to be associated with higher knowledge or confidence levels. Furthermore, levels of knowledge were not associated with confidence levels (Additional file 1: Table S4).

## Discussion

The study has shown that the knowledge of providers working in post-rape services was low, highlighting an urgent need to improve training in this field in the country. In particular, specific gaps in areas of knowledge need to be targeted in these training programmes. Knowledge and confidence was not associated with being trained previously which reflects poorly on the previous training received. Crucially, levels of knowledge were higher among doctors than nurses and those who had a more appropriate attitude towards rape, and perhaps were thus more motivated to learn. No previous studies on training for post-rape care including both doctors and nurses have been done, and neither were there studies found that assessed attitudes of providers in post-rape care services, and therefore these findings could not be compared to other studies. Knowledge seems to be critically dependant on attitude, which highlights the need for educating providers on rape and the seriousness of the problem. Confidence levels were much higher than knowledge, which is potentially dangerous; especially as the knowledge level regarded as ‘high’ here had a low threshold (50% or more).

The training programme included a range of providers from junior staff to those who were experienced. Yet there were still substantial gaps in the knowledge of providers and many providers were not completely confident in delivering every aspect of post-rape care. The study also found that providers who were previously trained did not have significantly greater levels of knowledge and confidence, but that confidence levels were disproportionately higher than levels of knowledge in all of the providers. This could be related to the manner in which training was offered in the country through a piecemeal approach, with poor or absent content, giving providers a sense of assurance in providing service yet in fact not improving knowledge on post-rape care.

In addition to levels of knowledge, provider attitudes when delivering services are important for patients [18,19]. Interestingly, providers with a more appropriate attitude towards rape had significantly greater odds of having high levels of knowledge. Studies have found that intimate partner violence and rape are not considered to be a health priority for providers [2,23,24], and some studies have found that where health care providers have a more sympathetic and appropriate attitude towards rape survivors, there is an indication of better clinical practices [25,26]. A previous study conducted in South Africa found that providers who perceived rape to be a serious medical problem provided a better quality of care [2]. However, no study has looked at the association of provider attitudes with knowledge. This is important as it impacts on decisions related to how providers should be trained, as attitudes are linked to improved knowledge and potentially better service provision.

Working in a facility with a crisis centre and being involved in the care of survivors was found to be significantly associated with higher levels of confidence. It has been reported that the experience of working with the same types of patients improves clinical skills [27] and therefore can lead to higher levels of confidence. Strengthening practical skills through the management of survivors and a supportive environment may also benefit providers working in crisis centres. It would be important to know how well providers working in crisis centres respond to further training to increase knowledge, in which case there would be evidence supporting this model to potentially offer better care.

However, it is still a matter of some concern that many providers do not have adequate levels of knowledge. Providers who had worked for more than 15 years in the same facility were found to have low levels of knowledge and similarly, older providers had a lower odds of having high knowledge levels. This indicates the need for continuous training and development of providers during the in-service period. There is a risk that providers become complacent and if they are not informed of current updates in the literature over time, they may not change their practices. As this discipline is constantly changing and new practices are being developed based on current evidence, there is always a need for continuous professional updates. Nurses were also found to have a lower odds of having high knowledge levels, and this is reflective of the current health service organization in South Africa, where the role of nurses in the provision of post-rape care has not been adequately defined. Thus nurses tend to receive minimal training and in most areas are only involved peripherally in the provision of post-rape care.

A high percentage of providers had experienced violence as victims or perpetrators, and startlingly a third of male providers had committed rape themselves. This highlights the importance of being extremely watchful for vicarious trauma, as affected providers are particularly vulnerable. There is also a need to understand the impact of having perpetrated violence against women on the care provided by male providers. This could be manifested through the provision of poor quality care with a lack of empathy, or could result in emotional problems for the provider through having to provide care for survivors. Exploration of this aspect was limited by the small sample size.

The generalizability of the findings in this study is limited, as some of the providers of the training were selected because they were known to be experts who would themselves later train, and this may have resulted in a bias towards a more knowledgable and confident group. The Western Cape Department of Health declined to participate in the pilot study and therefore there were no providers from this province included in the study. Some of the data were sensitive in nature but the questionnaires were self-administered to limit underreporting and ensure confidentiality. There was also a potential for recall bias. The knowledge and confidence measures were developed and used for the first time in this study. Although piloted on a small group of doctors prior to the study, these measures still need to be further assessed for reliability. Nine questions that were found to produce high levels of failure were dropped from the knowledge score. Providers did not respond to all questions in the questionnaire and this resulted in missing data for some of the variables. Providers were only asked about their provincial location and therefore no distinction can be made between providers working in urban and rural settings within the provinces. The small sample size limited further analysis of the data, for example, by sex of the doctors.

## Conclusions

The study indicates that there were gaps in knowledge even in providers who had been previously trained and that, although confidence was generally high, there were very poor levels of knowledge. A comprehensive standardized training curriculum for post-rape care services was undoubtedly needed in the country. It is clear that if the objectives of the National Sexual Assault Policy [1] and The Criminal Law (Sexual Offences and Related Matters) Amendment Act [28] are to be achieved; much has to be done to increase the number of providers who are trained on the management of post-rape care, to ensure that training is comprehensive, and to ensure that providers who are trained remain knowledgeable and skilled in current best practices. The development of this curriculum was critical and if found to result in improvements after the pilot training, this should then be expanded in a national training programme with scheduled refresher courses. Improving providers’ understanding of rape is important as this study has shown that providers who have a more appropriate attitude towards rape have higher levels of knowledge in providing post-rape care.

## Abbreviations

CHC: Community health centre; CI: Confidence interval; IPV: Interpersonal violence; OR: Odds ratio; SA: South Africa; SANE: Sexual assault nurse examiner; USA: United States of America.

## Competing interests

The authors declare that they have no competing interests.

## Authors’ contributions

RJ, RJ and NC were involved in the conceptualization and design of the study and the development of the data collection tools. LL and RJ were responsible for the collection and entry of data. RJ and RJ analysed the data and drafted the manuscript. All authors read, and approved the final manuscript.

## Authors’ information

RuxanaJ is a public health medicine specialist at School of Public Health, University of the Witwatersrand. RuxanaJ has an interest in women’s health, focusing specifically on gender-based violence and health. RachelJ is trained as a public health physician, epidemiologist and qualitative researcher. She is the Director of the Gender and Health unit at the Medical Research Council and Secretary of the global Sexual Violence Research Initiative. NC is a senior lecturer and coordinator of the Masters of Public Health programmes at the School of Public Health, University of the Witwatersrand. NC holds a PhD from the Rollins School of Public Health, Emory University. Over the last ten years, NC has worked on the development of gender-based violence services through policy formulation and research. LL is researcher at the Sexual Violence Research Initiative, hosted by the South African Medical Research Council. She holds a Masters Degree in Sociology with Specialization in Gender Studies.

## Pre-publication history

The pre-publication history for this paper can be accessed here:

http://www.biomedcentral.com/1472-6963/13/257/prepub

## Supplementary Material

Additional file 1: Table S1 Demographic and other background of health care providers. **Table S2.** Experience and training backgrounds of health care providers. **Table S3.** Knowledge by topic. **Table S4.** Regression models for factors associated with knowledge and confidence. Click here for file
