# Supplementary material for: Knowledge and confidence of South African health care providers regarding post-rape care: a cross-sectional study
Source: BMC Health Serv Res. 2013 Jul 3;13:257. doi: 10.1186/1472-6963-13-257 (PMC3733892; doi:10.1186/1472-6963-13-257)
Supplement: Additional file 1: Table S1 — Demographic and other background of health care providers. Table S2. Experience and training backgrounds of health care providers. Table S3. Knowledge by topic. Table S4. Regression models for factors associated with knowledge and confidence. [file 1472-6963-13-257-S1.doc]

Table 1. Demographic and other background of health care providers

|  | **Total** | **Knowledge** | | | **Confidence** | | |
| --- | --- | --- | --- | --- | --- | --- | --- |
|  | n (%) /  mean (SD) | **High (≥50%)**  n (%) /  mean (SD) | **Low (<50%)**  n (%) /  mean (SD) | **p value** | **High (≥80%)**  n (%) /  mean (SD) | **Low (<80%)**  n (%) /  mean (SD) | **p value** |
| **Sex (N=124)**  Male  Female | 37 (29.8%)  87 (70.2%) | 13 (44.8%)  16 (55.2%) | 23 (26.4%)  64 (73.6%) | 0.064 | 10 (30.03%)  23 (69.7%) | 21 (31.3%)  46 (68.7%) | 0.916 |
| **Age (N=123)** | 41.7 (8.8) | 37 (8.0) | 43 (8.0) | 0.002 | 40 (8.0) | 42 (10) | 0.462 |
| **Rank (N=123)**^*^  Doctor  Nurse | 39 (31.7%)  84 (68.3%) | 17 (60.7%)  11 (39.3%) | 21 (24.1%)  66 (75.9%) | 0.000 | 12 (36.4%)  21 (63.6%) | 24 (36.4%)  42 (63.6%) | 1.000 |
| **Province (N=124)**^#^  Eastern Cape  Free State  Gauteng  KZN  Limpopo  Mpumalanga  North West  Northern Cape | 20 (16.1%)  13 (10.5%)  15 (12.1%)  20 (16.1%)  15 (12.1%)  13 (10.5%)  13 (10.5%)  15 (12.1%) | 2 (6.9%)  6 (20.7%)  3 (10.3%)  8 (27.6%)  3 (10.3%)  3 (10.3%)  0 (0.0%)  4 (13.8%) | 17 (19.5%)  4 (4.6%)  12 (13.8%)  12 (13.8%)  12 (13.8%)  9 (10.3%)  12 (13.8%)  9 (10.3%) | 0.025 | 4 (12.1%)  8 (24.2%)  6 (18.2%)  3 (9.1%)  2 (6.1%)  7 (21.2%)  0 (0.0%)  3 (9.1%) | 10 (14.9%)  5 (7.5%)  8 (11.9%)  11 (16.4%)  9 (13.4%)  5 (7.5%)  7 (10.5%)  12 (17.9%) | 0.034 |
| **IPV (N=124)** ^#^  None  Experienced IPV (female providers only)  Perpetrated IPV (male providers only) | 64 (51.6%)  47 (37.9%)  13 (10.5%) | 14 (48.3%)  10 (34.5%)  5 (17.2%) | 47 (54.0%)  33 (37.9%)  7 (8.1%) | 0.406 | 14 (42.4%)  15 (45.5%)  4 (12.1%) | 34 (50.8%)  24 (35.8%)  9 (13.4%) | 0.639 |
| **Rape attitude score (N=94)** | 65.9 (8.4) | 69.0 (7.2) | 64.5 (8.7) | 0.027 | 68.1 (6.6) | 66.4 (8.6) | 0.371 |
| **Gender attitude score (N=99)** | 69.5 (7.1) | 70.5 (5.9) | 68.8 (7.4) | 0.269 | 71.3 (5.5) | 69.2 (7.8) | 0.189 |
| **Empathy score (N=111)** | 16.2 (3.6) | 16.2 (3.7) | 16.3 (3.6) | 0.870 | 16.7 (4.0) | 16.1 (3.7) | 0.472 |

Sample sizes vary due to missing data.

^*^One doctor sessional, three nurses in administrative roles: managerial/coordinators

^#^ Fisher Exact test

Table 2. Experience and training backgrounds of health care providers

|  | **Total** | **Knowledge** | | | **Confidence** | | |
| --- | --- | --- | --- | --- | --- | --- | --- |
|  | n (%) /  mean (SD) | **High (≥50%)**  n (%) /  mean (SD) | **Low (<50%)**  n (%) /  mean (SD) | **p value** | **High (≥80%)**  n (%) /  mean (SD) | **Low (<80%)**  n (%) /  mean (SD) | **p value** |
| **Type of facility (N=121)**^#^  Clinic/CHC^*^  District hospital  Regional hospital  Tertiary hospital | 39 (32.2%)  53 (43.8%)  19 (15.7%)  10 (8.3%) | 7 (25.0%)  13 (46.4%)  5 (17.9%)  3 (10.7%) | 31 (36.5%)  35 (41.2%)  13 (15.3%)  6 (7.1%) | 0.661 | 11 (33.3%)  16 (48.5%)  5 (15.2%)  1 (3.1%) | 20 (30.8%)  30 (46.2%)  9 (13.9%)  6 (9.2%) | 0.798 |
| **Crises centre in facility (N=124)** | 55 (44.4%) | 16 (55.2%) | 34 (39.1%) | 0.130 | 22 (66.7%) | 23 (34.3%) | 0.002 |
| **Total time in service (N=120)**^#^  <10  10 – 19  ≥20 | 35 (29.2%)  47 (39.2%)  38 (31.7%) | 14 (50.0%)  9 (32.1%)  5 (17.9%) | 21 (25.0%)  36 (42.9%)  27 (32.1%) | 0.054 | 10 (30.3%)  13 (39.4%)  10 (30.3%) | 21 (32.3%)  25 (38.5%)  19 (29.2%) | 1.000 |
| **Service time at current facility (N=119)**^#^  <10  10 – 14  ≥15 | 72 (60.5%)  26 (21.9%)  21 (17.7%) | 20 (74.1%)  7 (25.9%)  0 (0.0%) | 50 (59.5%)  18 (21.4%)  16 (19.1%) | 0.030 | 23 (71.9%)  6 (18.8%)  3 (9.4%) | 41 (65.1%)  10 (15.9%)  12 (19.1%) | 0.520 |
| **Examined survivor and completed J88 in last 3 months (N=123)** | 61 (49.6%) | 21 (72.4%) | 37 (42.5%) | 0.005 | 24 (72.7%) | 29 (43.3%) | 0.006 |
| **Proportion of survivors seen who come to facility (N=116)** ^#^  <20%  21% – 40%  41% - 60%  61% - 100% | 44 (37.9%)  20 (17.2%)  27 (23.3%)  25 (20.5%) | 11 (37.9%)  7 (24.1%)  5 (17.2%)  6 (20.7%) | 37 (43.5%)  13 (15.3%)  19 (22.4%)  16 (18.8%) | 0.694 | 8 (24.2%)  4 (12.1%)  9 (27.3%)  12 (36.4%) | 33 (49.3%)  10 (14.9%)  15 (22.4%)  9 (13.4%) | 0.027 |
| **Country trained undergrad**^#^  SA  Elsewhere | 106 (86.2%  17 (13.8%) | 24 (82.8%)  5 (17.2%) | 76 (87.4%)  11 (12.6%) | 0.542 | 30 (90.1%)  3 (9.1%) | 55 (83.3%)  11 (16.7%) | 0.374 |
| **Trained on counselling (N=124)** | 98 (79.0%) | 25 (86.2%) | 68 (78.2%) | 0.429 | 29 (87.9%) | 53 (79.1%) | 0.408 |
| **Trained on post-rape care (N=120)** | 72 (59.5%) | 20 (69.0%) | 46 (54.8%) | 0.181 | 26 (78.8%) | 35 (53.0%) | 0.013 |
| **High knowledge (≥50%) (N=116)** | 29 (25.0%) |  |  |  |  |  |  |
| **High confidence (≥80%) (N=100)** | 33 (33.0%) | 9 (33.3%) | 20 (30.8%) | 0.810 |  |  |  |

Sample sizes vary due to missing data.

^*^ CHC: Community health centre; ^#^ Fisher Exact test

Table 3. Knowledge by topic

| **Topic** | **Number of questions** | **Percentage median score (Interquartile range)** |
| --- | --- | --- |
| Context of sexual assault in south Africa | 6 | 50% (0 – 83.3%) |
| Laws and court processes | 6 | 50% (16.7 - 83.3%) |
| Sexual rights  E.g. Emma describes herself as transgendered. Which one of the following definitions most accurately describes what the term “transgendered”?   1. She is romantically and sexually attracted to both women and men 2. She is confused about her sexual orientation and thinks she might be attracted to women 3. She is a person born with a combination of male and female reproductive organs 4. She was born with a male body but has started sexual reassignment surgery so that her body matches her gender identity | 5 | 60% (0 – 100%) |
| Communication  E.g. Which of the following is an example of good listening?   1. “So in summary, you were hijacked while on the way home from work….” 2. “Did you say that you were from Westdene? My grandparents live there” 3. “Do not worry about the court case. Everything will be fine” 4. “Let me tell you what to do” | 3 | 33.3% (0 – 100%) |
| Provision of medical care | 4 | 50% (0 – 100%) |
| Examination and evidence collection of adult sexual assault survivors | 8 | 25% (0 – 75%)) |
| Management of sexually assaulted children  E.g. Which one of the following is definitely indicative of assault in a child?   1. An absent posterior fourchette 2. A hymenal bump 3. A posterior attenuated hymen 4. An anal tag | 6 | 33.3% (0 – 83.3%) |
| Mental health care | 9 | 33.3% (0 – 55.6%) |
| Prevention and management of pregnancy post-rape  E.g. A 22 year old women presents to the health care facility. She has been raped by a stranger last night. Her last menstruation was three weeks ago and she is medically well. The emergency contraceptive regimen of choice for her is:   1. Norlevo 2 tabs (levonorgestrel 1500μg) stat po 2. Ovral 4 tabs (ethinyl estradiol 100μg/levonorgestrel 500μg) stat po 3. Nordette 2 tabs (ethinyl estradiol 125μg/levonorgestrel 300μg) stat po 4. Triphasil 4 tabs (ethinyl estradiol 125μg/levonorgestrel 500μg) stat po | 6 | 33.3% (16.7 – 83.3%) |
| Prevention and management of infectious diseases | 4 | 50.0% (0 – 75%) |
| Prevention and management of HIV post-rape  E.g. A patient presents 12 hours after being gang raped. Pre-test HIV counselling is carried out but she refuses to have an HIV test  What should the health care provider do?   - 1. Refuse to give her post exposure prophylaxis   2. Ask the patient to return in 3 days so that she can be pre-test counseled again   3. Provide a 3 day supply of post exposure prophylaxis and ask the patient to come back in 3 days and pre-test counsel again   4. Provide a 7 day supply of post exposure prophylaxis and ask the patient to come back every week for a month for post exposure prophylaxis so that she will have three more opportunities to be pre-test counseled | 4 | 50% (0 – 100%) |
| Documentation | 2 | 50% (0 – 100%) |
| Follow-up | 1 | 100% (0 – 100%) |
| Vicarious trauma  E.g. Countertransference occurs when:   - 1. Health care providers experience symptoms of trauma themselves from work with trauma survivors   2. Health care providers bring into their work with patients aspects of their own past history   3. Health care providers become very judgmental and unsympathetic   4. Health care providers working with trauma survivors develop burn out | 3 | 33.3% (0 – 100%) |

**Table 4. Regression models for factors associated with knowledge and confidence**

|  | **Knowledge** | | | | **Confidence** | | | |
| --- | --- | --- | --- | --- | --- | --- | --- | --- |
|  | **Univariate** | **p value** | **Multivariate** | **p value** | **Univariate** | **p value** | **Multivariate** | **p value** |
| **Sex**  Male  Female | -  0.44 (0.18 – 1.06) | 0.067 |  |  | -  1.05 (0.43 – 2.59) | 0.916 |  |  |
| **Age** | 0.91 (0.86 – 0.97) | 0.003 | 0.94 (0.88 – 1.00) | 0.053 | 0.98 (0.94 – 1.03) | 0.458 |  |  |
| **Rank**  Doctor  Nurse | -  0.45 (0.29 – 0.71) | 0.001 | -  0.40 (0.23 – 0.69) | 0.001 | -  1.00 (0.65 – 1.54) | 1.000 |  |  |
| **Province**  Gauteng  Mpumalanga  North West  Limpopo  KZN  Northern Cape  Eastern Cape  Free State | -  1.33 (0.22 – 8.22)  -  1.0 (0.17 – 5.99)  2.67 (0.57 – 12.57)  1.78 (0.32 – 10.01)  0.47 (0.07 – 3.27)  6.0 (1.00 – 35.91) | 0.757  1.000  0.215  0.514  0.445  0.050 |  |  | -  1.87 (0.39 – 8.89)  -  0.30 (0.05 – 1.91)  0.36 (0.07 – 1.91)  0.33 (0.06 – 1.73)  0.53 (0.11 – 2.56)  2.13 (0.46 – 9.94) | 0.433  0.200  0.232  0.192  0.433  0.335 |  |  |
| **IPV**  None  Experienced IPV (female providers)  Perpetrated IPV (male providers) | -  1.65 (0.68 – 4.00)  3.88 (1.10 – 13.77) | 0.269  0.036 |  |  | -  1.52 (0.62 – 3.72)  1.08 (0.28 – 4.09) | 0.362  0.911 |  |  |
| **Rape attitude score** | 1.08 (1.01 – 1.15) | 0.030 | 1.10 (1.01 – 1.19) | 0.026 | 1.03 (0.97 – 1.09) | 0.368 |  |  |
| **Gender attitude score** | 1.04 (0.97 – 1.11) | 0.267 |  |  | 1.05 (0.98 – 1.12) | 0.189 |  |  |
| **Empathy score** | 0.99 (0.88 – 1.11) | 0.869 |  |  | 1.05 (0.93 – 1.18) | 0.469 |  |  |
| **Type of facility^*^**  Clinic/CHC  District hospital  Regional hospital  Tertiary hospital | -  1.64 (0.58 – 4.65)  1.70 (0.46 – 6.36)  2.21 (0.44 – 11.08) | 0.347  0.428  0.333 |  |  | -  0.97 (0.37 – 2.52)  1.01 (0.27 – 3.77)  0.30 (0.03 – 2.85) | 0.950  0.988  0.296 |  |  |
| **Crises centre in facility**  No  Yes | -  1.92 (0.82 – 4.48) | 0.133 |  |  | -  3.83 (1.58 – 9.24) | 0.003 | -  3.06 (1.18 – 7.93) | 0.021 |
| **Total time in service**  <10  10 – 19  ≥20 | -  0.38 (0.14 – 1.01)  0.28 (0.09 – 0.89) | 0.053  0.032 |  |  | -  1.09 (0.40 – 2.99)  1.11 (0.38 – 3.24) | 0.864  0.855 |  |  |
| **Service time at current facility**  <10  10 – 14  ≥15 | -  0.97 (0.35 – 2.68)  - | 0.957 |  |  | -  1.07 (0.34 – 3.32)  0.45 (0.11 – 1.74) | 0.907  0.246 |  |  |
| **Examined survivor and completed J88 in last 3 months**  No  Yes | -  3.55 (1.42 – 8.89) | 0.007 |  |  | -  3.49 (1.41 – 8.64) | 0.007 | -  3.30 (1.24 – 8.81) | 0.017 |
| **Proportion of survivors seen who come to facility**  <20%  21% – 40%  41% - 60%  61% - 100% | -  1.81 (0.58 – 5.66)  0.89 (0.27 – 2.92)  1.26 (0.40 – 4.00) | 0.307  0.841  0.693 |  |  | -  1.65 (0.41 – 6.65)  2.48 (0.80 – 7.67)  5.5 (1.73 – 17.53) | 0.481  0.116  0.004 | -  1.33 (0.30 – 6.01)  2.26 (0.68 – 7.52)  4.31 (1.23 – 15.10) | 0.709  0.185  0.022 |
| **Country trained undergrad**  SA  Elsewhere | -  1.44 (0.45 – 4.56) | 0.536 |  |  | -  0.50 (0.13 – 1.93) | 0.315 |  |  |
| **Trained on counseling**  No  Yes | -  3.62 (1.39 – 9.41) | 0.008 |  |  | -  1.92 (0.58 – 6.36) | 0.298 |  |  |
| **Trained on post-rape care**  No  Yes | -  1.84 (0.75 – 4.50) | 0.184 |  |  | -  3.29 (1.25 – 8.63) | 0.016 |  |  |
